# Synergestic Protein-Green synthesized Nanoparticles Nanosystems: A Sustainable and Safe Approach for Cancer Theranostics

**DOI:** 10.7150/ntno.118766

**Published:** 2025-10-24

**Authors:** Ashitha Washington, Gopal Shankar Krishnakumar, Ravindra Kumar

**Affiliations:** Computational Biology and Bioinformatics Lab, Department of Bioscience and Engineering, National Institute of Technology Calicut, Kozhikode, Kerala, India, 673601.

**Keywords:** Cancer theranostics, Nanomaterials, Nanoparticles, Nanostructures, Nanosystems

## Abstract

**Rationale:** Cancer theranostics is an evolving field focused on reducing mortality and providing safer treatment options for complete remission. The rising incidence of cancer and increased mortality linked to frequent hospital visits highlight the need for nature-based remedies as promising alternatives. Nanotechnology has contributed to cancer treatment by offering effective anti-cancer and antimicrobial solutions, with a current emphasis on developing safe, easily synthesized nanomaterials using natural sources and green synthesis methods.

**Methods:** This review explores the synergistic use of protein-based nanomaterials and green-synthesized nanoparticles in cancer theranostics. Sources of protein-based nanomaterials include human serum albumin, gliadin, DNA, peptides, collagen, bacteria, and soy protein. Green-synthesized nanoparticles discussed include gold, silver, copper, zinc, and magnesium. The approach involves evaluating the stability, biocompatibility, and therapeutic potential of these nanosystems based on existing experimental findings.

**Results:** Protein-based nanomaterials and green-synthesized nanoparticles demonstrate synergistic effects that enhance their stability and efficacy in cancer theranostics. These nanosystems offer anti-cancer activity along with additional functional properties resulting from their synergistic composition. Furthermore, they are environmentally friendly and non-toxic. Despite their promise, the literature reveals a gap in studies investigating these hybrid nanosystems, particularly regarding *in vivo* evaluations.

**Conclusions:** Synergistic protein-green synthesized nanoparticle nanosystems hold significant promise for cancer theranostics due to their enhanced therapeutic properties and environmental safety. However, additional *in vivo* studies are crucial to fully establish their efficacy. Future research should leverage emerging technologies to accelerate the development and testing of stable nanosystems for clinical application.

## Introduction

Cancer is an umbrella term that refers to the proliferation and unrestricted development of abnormal cells within various body parts and systems, including the blood, skin, digestive, urinary, respiratory, skeletal, reproductive, and neural systems 1,2. Cancer remains one of the most lethal diseases in the world, with new cases increasing year after year 3. International Agency for Research on Cancer reports estimates that one in every four deaths due to non-communicable diseases is caused due to cancer and an estimated of 20 million new cases were reported in 2022 alone 4,5. The report also estimates that approximately half of the diagnosed cases would die, making the mortality rate almost 50 percent 6. The estimates are expected to be much higher as there are data insufficiencies due to lack of data from some countries, especially from low income and middle-income countries; data inconsistencies during data collection are another reason 6. Research about tumor biology along with advancements in diagnostic techniques and anti-cancer therapies has contributed a significant 29% reduction in cancer-related mortality between 1991 and 2017 3. Current standard treatment plan for metastatic cancer includes usage of pharmaceutical chemotherapeutic agents, surgical resection, radiation and targeted therapy on growth factors 7. The remedies, especially pharmaceutical chemotherapeutic drugs are often more expensive than natural alternatives, limiting their accessibility, especially in resource-constrained settings 2. Cancer patients from low- and middle-income countries often resort to risky financial decisions due to the burden of high medication costs, repeated visits, and palliative care 8,9. Despite advances in cancer treatment, current strategies frequently cause more harm than good, with high recurrence rates persisting even after surgery and medication 10. A vital component of anti-cancer treatment is chemotherapy 11. Nevertheless, a critical issue known as “drug resistance” has emerged in the realm of cancer chemotherapy 12. Frequent exposure to chemotherapeutic agents can render cancer cells unresponsive to their inhibitory effects 12. To counteract this challenge, high doses of these agents are administered to impede cancer progression. However, the use of elevated doses has a drawback - it leads to dose-dependent side effects, imposing limitations 12. Most of the second- and third-stage cancer patients often fail to meet the inclusion criteria for clinical studies because they are heavily medicated and at risk for multi-drug resistance 13. Multiple anti-cancer drug resistance accounts for approximately 9 in every 10 cancer-related deaths 13. Also, there is higher clinical complexity for cancer patients compared to non-cancer patients as the patients often require frequent admissions to acute care hospitals 14. The length of stay and readmission rates are higher for cancer patients compared to non-cancer patients, making them highly exposed to the pathogen infection, causing bloodstream infections, often ending with sepsis 14,15. Infections are mostly caused by Gram-negative bacteria 15, thus generating more interest in developing cancer theranostics with dual properties that can work for both bacterial infections as well as for the cancer, as seen in case of lung cancer patients being administered boron and zinc derived nanomaterials for their anti-bacterial properties 16. Nanotechnology has been increasingly viewed as a game-changer, with as many as around 75 anti-cancer formulations in clinical trials, with approximately more than 15 of them FDA approved 17. However, there is still ongoing research as there are many nanoformulations that have not received approval for clinical application due to their toxicity in tissues and difficulties in obtaining the ideal nano-formulation size for optimum anti-cancer activity; with additional properties such as anti-bacterial activities also being needed in combinatorial therapies for different types of cancer 17.

The war on cancer is far from being won, and the evolution of cancer theranostics depends on our ability to innovate, drawing inspiration from both modern science and nature 13. Cancer theranostics combines diagnosis and therapy to enhance personalized cancer care by streamlining treatment, reducing durations, and improving outcomes through early intervention during diagnosis 18. The purpose of this review is to bring together nanomaterials that can be used for sustainable cancer theranostics. Protein based nanomaterials are majorly known for their non-toxicity and green synthesized nanoparticles are known to be highly efficient in low dosages. Together they can be considered for safe and environmentally friendly cancer treatment that can potentially add more years to the cancer prognosis. The new age of cancer theranostics would benefit from harnessing the synergestic effect of the hybrid nanosystems comprising of proteins and green synthesized nanoparticles. The principles behind their synergy are chemical bonding, self-assembly, *in-situ* incorporation and adsorption 19-21. Their synergestic effect offers better performance in properties 22 such as tumor imaging, diagnosis, drug delivery and controlled drug release; all of which is necessary for a biocompatible cancer theranostics 23. Additionally, the side-effects due to microbial infection can be combated with the combinatorial approach of these nanomaterials. This review describes each nanomaterial's potential and application, overall limitations and possible combinatorial approaches, that can bring together possible candidates to develop sustainable cancer theranostics as appropriate.

## Harnessing synergistic nanoparticles from nature

Naturally derived remedies have stood the test of time, with approximately 85% of the global population relying on them 24. Their affordability, minimal side effects, and ease of preparation make them highly valuable across diverse geographic and socioeconomic communities 24. Furthermore, these remedies pose no significant risks to human health or the environment 25.

In recent years, there has been a growing preference for naturally synthesized nanomaterials, primarily due to their cost-effectiveness, safety, and low systemic toxicity 17. Nature offers a vast arsenal of proteins and extracts from plants and animals, which can be harnessed to create anti-cancer nanoscale molecules with high functional synergy. The scientific community has shown increasing interest in hybrid nanostructures combining proteins and nanoparticles 26. Four key properties: shape, size, charge, and surface functionalization—collectively determine how a protein will “experience” or interact with a given nanoparticle, significantly influencing protein adsorption, conformational changes, and biological responses 26. Preliminary studies suggest that when nanoparticles come into contact with proteins, a corona layer forms around them, enhancing their stability 26. Strong interactions are observed in colloidally stable nanosystems, where proteins contribute to nanoparticle stabilization 26. Recent research has highlighted the role of hydrophobic forces, hydrogen bonding, and van der Waals forces in facilitating these interactions 27. Human serum albumin has demonstrated remarkable stability as a coating protein, with hybrid nanoparticles maintaining stability in water, Dulbecco's phosphate-buffered saline, and Dulbecco's modified Eagle medium 26. Several studies have supported the viability of hybrid nanosystems involving human serum albumin and metals such as silver, copper, iron, zinc, and even rare metals like niobium 26-30. The use of protein nanostructures as carriers for green nanoparticles presents promising opportunities for sustainable and safe cancer theranostics. Plant molecular farming enables plants to produce proteins, expanding the scope for combinatorial approaches 31. Under optimal conditions, metal nanoparticles and protein nanostructures can self-assemble to form hybrid multimeric nanomaterials 32. In these hybrids, proteins often serve as both linking and reducing agents for the metal nanoparticles 33. This synthesis is achievable through protein-nanoparticle co-engineering, leveraging the self-assembly principle of biological systems 34,35. Self-assembly involves intermolecular interactions such as van der waals forces 36, Hydrogen bonding 37, Ion-dipole interactions 38,39, Ion-Induced dipole interactions 40, and Dipole-induced dipole interactions 19. Inorganic materials can be combined into organic materials while synthesizing, through *in-situ* incorporation 20. The materials can also be adsorped, which makes the amalgamation of the nanomaterials easier 20.

Although the available literature is still in its early stages, more *in vitro* and *in vivo* studies are needed to fully explore the biomedical applications of these hybrid nanosystems. Complementary research investigating their additional properties will serve as a foundation for developing novel hybrid nanostructures. For instance, a study by Rezazadeh et al. (2020) demonstrated enhanced functional properties of silver nanoparticles when combined with chitosan-algae extract through biosynthesis. The synergy between the polysaccharide-based extract and nanoparticles not only controlled the size of the silver nanoparticles but also increased their bioavailability 41. Furthermore, for effective antimicrobial activity, nanomaterials must either directly interact with microbes or facilitate the entry of antimicrobial agents 42. Characterization studies of green-synthesized nanoparticles have shown promising antimicrobial properties, with high stability even without capping agents 43. Notably, silver nanoparticles combined with bacteriophages (which consist of nucleic acid enclosed in a protein coat) have demonstrated efficacy in preventing secondary infections caused by *Salmonella*, a Gram-negative bacterium 44. Gliadin based silver nanoparticles have been assessed for their anti-bacterial properties, which can be also extended to anti-cancer studies 45.

Studies have also shown that plant-derived silver nanoparticles exhibit superior antimicrobial activity compared to plant extracts alone 46. Additionally, silver nanoparticles conjugated with albumin, collagen, zein, and lysozyme have demonstrated remarkable drug cargo uptake by osteosarcoma cell lines 47. Small molecules, proteins, peptides, and nucleic acid sequences can be modified through mosaic, vertex, capsule, or cantilever approaches to function as effective carriers for diverse anti-cancer nanoparticles, including metals 48. Furthermore, silver nanoparticles conjugated with Hepatitis B core antigen protein via an *in vivo* asymmetric self-assembly strategy could be used to develop immunoassays for diseases, including cancer 49. Zinc-Chitosan nanoparticles exhibited stronger antibacterial effects, with minimal inhibitory concentrations of 9.25-13.5 µg/mL, completely inhibiting *Staphylococcus aureus* and *Escherichia coli* 50. In anticancer activity, Zinc-Chitosan nanoparticles triggered apoptosis in human acute T-lymphocyte leukemia cells, leading to 65-70% cellular damage 50. The results suggest that Zn-CSNPs hold promise as a therapeutic approach for treating zinc-deficiency-related diseases, particularly human acute leukemia 50.

Early studies have also demonstrated the cytotoxic potential of gold nanoparticles stabilized by gelatin for delivering adriamycin to leukemia cells 51. Drug internalization has been confirmed in gold-silica nanoparticles conjugated with human serum albumin, loaded with Doxorubicin, and sealed with Rose Bengal, creating a multifunctional nanosystem for diagnostics and therapeutics 52. An *in vivo* study further validated the potential of gold-human serum albumin conjugates as a controlled drug delivery system for metastatic colorectal cancer 53.

These findings collectively highlight the immense potential of hybrid nanosystems in biomedical applications, particularly in cancer therapy. However, further research is essential to refine their design and ensure their safety and efficacy in clinical applications.

## Protein based nanostructures

**Figure 1:**
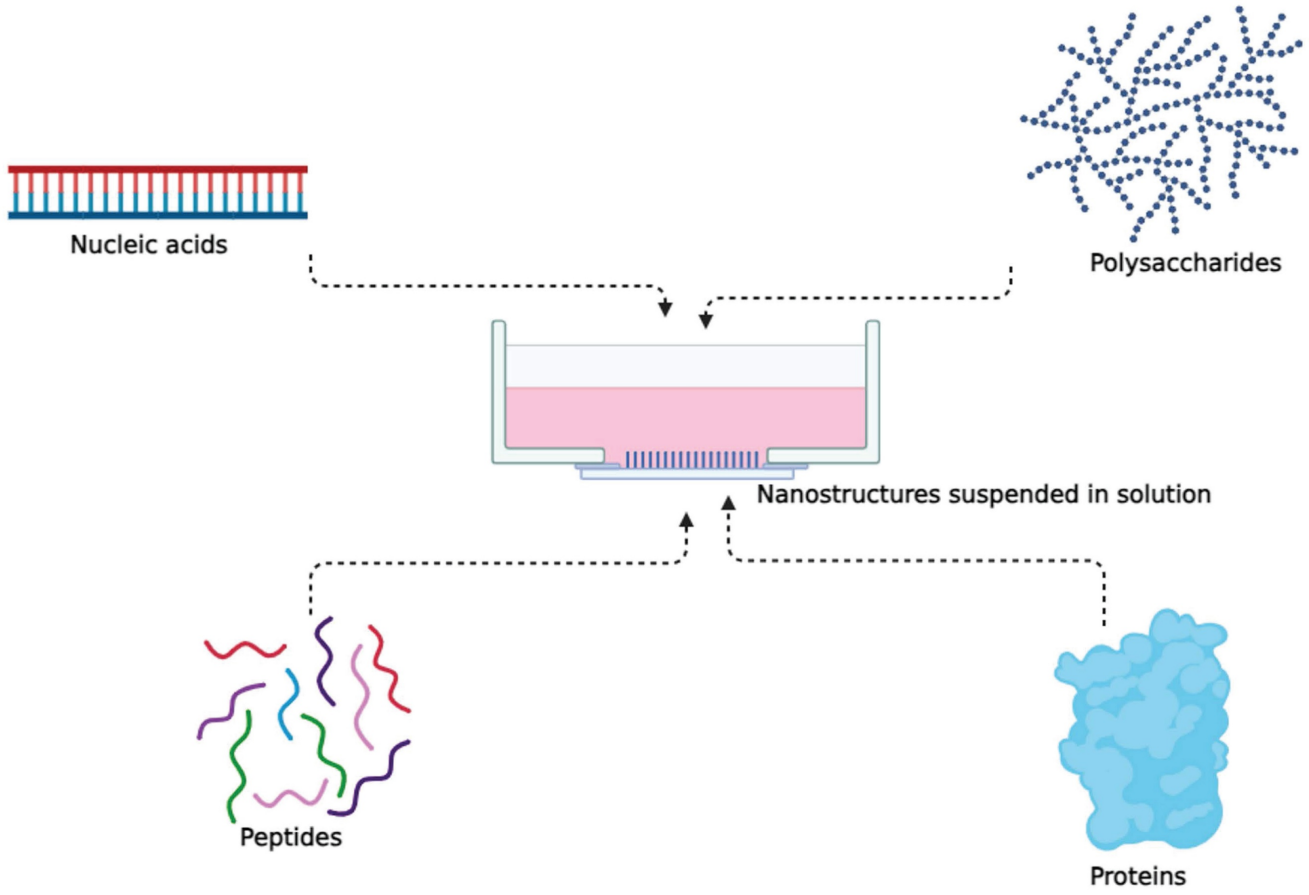
Nanostructures formed from simple to complex proteins.

There are many ways we can describe the existence of living beings. In one such narrative, proteins are the building blocks of all living beings 54. Protein structures can vary from simple monomeric proteins to more complex arrangements like oligomers, polymers, and networks formed through interactions between proteins 55,56** (Figure 1)**. It is not a surprise that proteins can be used in a beneficial way, such as, a part of cancer treatment process, in form of protein nanostructures 57.

**Figure 2:**
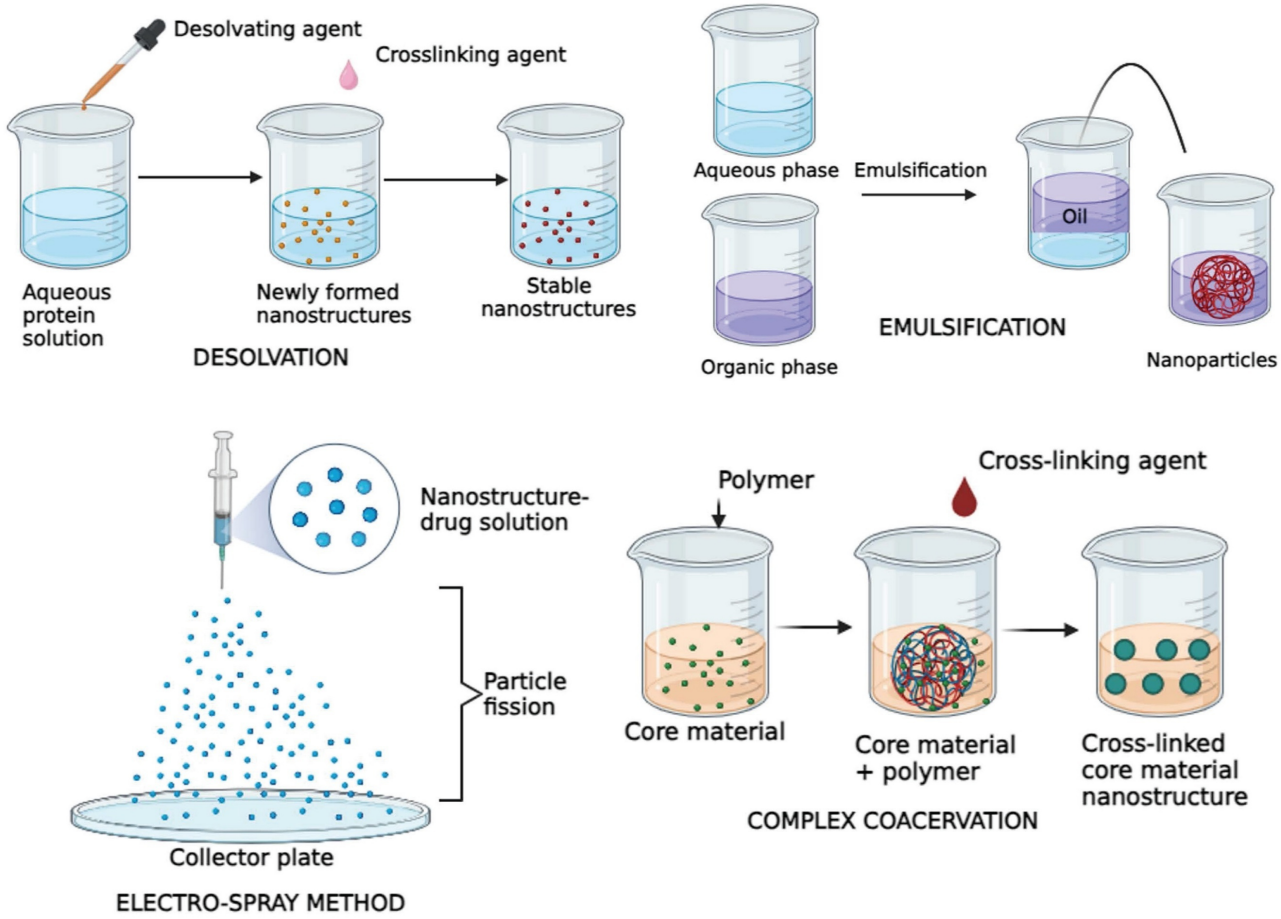
Illustration depicting major types of protein nanostructure synthesis methods.

Proteins are abundant in nature, which makes it easier for procurement and synthesis 58,59. A lot of these protein nanostructures are made using substances such as Albumin, Collagen, Gelatin, Legumin, Elastin, Ferritin, Soybean, Milk protein, Zein and Gliadin 60,61. Although the raw materials involved are proteins, they are reinforced to be structurally stable and customized to function, giving the desirable effect 58,59. The protein nanostructures are synthesized using techniques such as desolvation, emulsification, electrospray method and complex coacervation 62,61 **(Figure 2)*.*** The choice of preparation methods, site-specific modifications, and recombinant engineering for these nanostructures is influenced by the physicochemical properties and composition of the therapeutic agents and proteins 61.

Both naturally occurring and synthetic biomolecules, such as polysaccharides, nucleic acids, peptides, and proteins, have been investigated for the purpose of creating nanostructures in the field of cancer nanomedicine 63,64. Tetrahedral DNA nanostructures (TDNs) have gained global attention for their stability, biocompatibility, and ease of modification, making them versatile carriers for various therapeutic agents and imaging probes in drug delivery, molecular diagnostics, and biological imaging 65.

Nature has evolved a diverse repertoire of protein nanostructures over millions of years, equipping them with a multitude of functions 55. For example, viral capsids are a striking example of this phenomenon, as they are constructed from multiple copies of a monomeric protein unit, forming sturdy polyhedral structures crucial for safeguarding and transporting genetic material 66. Similarly, bacterial exotoxins like botulinum toxins consist of distinct structural domains, each serving a specific function, collectively making them potent weapons in the natural world 67. What adds to the intrigue is that post-translational modifications can further enhance the diversity of protein building blocks, expanding the array of functional nanostructures 57.

The development of well-defined nanostructures with bioactivity and favorable material properties is particularly significant in biomedicine, where stability, biocompatibility, biodegradability, functionality and biosafety are of paramount importance 68-70.

### Protein nanostructures in cancer therapeutics

Protein nanostructures are catalysts for multiple complex cellular mechanisms, through their function, molecular recognition and stable structural frameworks 69. Stability is very important, as they should never degrade in the host environment, which in this context would be the body of the cancer patient 71. Whether it is to deliver drugs to the site of tumor or to induce a cellular process, structural stability is there in protein nanostructure 72,73.

At lower doses, protein nanostructures perform well and there are usually lesser chemical reactions 62. Protein nanostructures also provide a solution to alleviate the side effects associated with anti-cancer therapy such as chemotherapy 74. They achieve this by selectively homing in on tumor cells, leaving healthy cells unharmed, thereby enhancing therapeutic effectiveness 75. Human serum albumin, one of the most common protein nanostructures used to deliver nanoparticles, is reported to enhance the anti-cancer and anti-bacterial properties, with least toxicity 76.

Protein nanostructures offer significant advantages in biomedical applications due to their remarkable multifunctionality. Their adaptable design allows for the integration of various functionalities within a single platform, making them particularly valuable for addressing complex diseases like cancer 77. These functionalities include targeted drug delivery, sequential targeting, responsiveness to stimuli, theranostics (combining therapy and diagnostics), combination treatments, and the incorporation of logic gates for precise therapeutic actions 61.

#### Human serum albumin

Human Serum albumin, the most abundant protein in the blood, is a potential candidate for medication administration. It has a half-life of 19 days 78. Through GP60-receptor-mediated transcytosis, it specifically binds to the secreted protein acidic and rich in cysteine, enabling it to efficiently enter vascular endothelial cells and selectively accumulate inside cancer tissues 79. According to Wang et al. (2021), via *in vivo* investigations, it has been shown that a nanovaccine composed of endogenous human serum albumin and specific constituents enhances both innate and adaptive immunity against several types of cancer. Albumin nanocarriers offer numerous advantages, including non-toxicity, biodegradability, ease of preparation, non-immunogenicity, precise sizing, and the presence of reactive groups like thiols, amines, and carboxyl groups 80. Various albumin-based cancer nanomedicines derived from anticancer formulations have been implemented in clinical trials, demonstrating the promise of albumin in treating cancer. The FDA has approved Abraxane, a nanomedicine that uses albumin as its basis, for the treatment of advanced breast, lung, and pancreatic cancer 61.

Saleh et al. utilized the desolvation method to craft human serum albumin (HSA) nanoparticles containing curcumin, which were then delivered to HER-2 positive breast cancer cells 80. The drug-loading efficiency was 3.4%, with an encapsulation efficiency of 71.3%, leading to enhanced stability and solubility of curcumin. Surface modification enabled targeted delivery by conjugating HER2 Apt to the nanoparticle surface 80. *Ex vivo* experiments confirmed that these albumin-loaded nanocarriers improve drug release, increased bioavailability, enhanced pharmacokinetic properties, and improved drug targeting to tissues 80. The use of HSA nanoparticles as vectors to transfer genes or antibodies has also been investigated. In a study, Mesken et al. used HSA nanoparticles coupled with a cell-penetrating peptide (CPP) to transfect HEK 293T cells. These nanoparticles were also generated using the desolvation process, with particle sizes ranging from 207.8 ± 21.3 to 222.8 ± 42.4 nm 81. Plasmid loading efficiency was confirmed *in vitro* to be unaffected by CPP surface alteration, with a value of 78.3 ± 13.0% 81. At high plasmid concentrations, transfection efficiency rose by up to 50% 81. Furthermore, HSA nanoparticles can be used as carriers of non-DNA payloads due to their low cytotoxicity. For instance, Redín et al. created HSA nanoparticles that were loaded with the chemical medication bevacizumab, which is used to treat certain eye illnesses and tumours 82. The resultant bevacizumab showed high stability and a two-phase release pattern, with a slower, continuous release occurring for more than 24 hours after an initial release of roughly 400 μg/mL during the first 5 minutes. Crucially, *in vivo* investigations verified the albumin nanoparticle's non-toxicity and showed mucosal adherence 62. It is important to note that albumin nanoparticles and other nanostructures produced from natural proteins have been used in medicinal applications in the past due to their inherent properties such as drug encapsulation, targeted delivery, stimulus-responsive conformational changes, synergistic theranostics, and enzymatic performances 83.

#### Gliadin

Gliadin is a gluten protein produced from wheat, showing promise as a polymer for oral and topical drug delivery systems 84. It is often used in mucoadhesive formulations because of its ability to adhere to mucous membranes. Gliadin offers several appealing attributes, including biocompatibility, biodegradability, natural origin, non-toxicity, and stability, making it an excellent candidate for drug delivery systems 84.

There have been few studies that conducted studies involving gliadin nanostructures loaded with anticancer drugs, performing *in vivo* experiments that induced apoptosis in breast cells 85, with combination of gelatin 86. Using an electrospray deposition method, their study was centred on creating gliadin and gliadin-gelatin composite nanostructures for the regulated release of the anticancer medication cyclophosphamide. Gliadin nanostructures containing cyclo-phosphamide exhibited a 48-hours release pattern, while gliadin-gelatin nanostructures exhibited faster release kinetics 85. Breast cancer cell cultures treated with cyclo-phosphamide-loaded gliadin nanostructures were maintained for 24 hours, resulting in cell apoptosis 85.

#### DNA

Because of its unique benefits and remarkable biocompatibility, co assembling proteins and DNA to form hybrid nanostructures is a rapidly expanding topic of study. When it comes to creating a variety of nanostructures, DNA is more precise than other nanomaterials. Conversely, proteins offer a multitude of well-established specific biological functions 87. Consequently, DNA-protein nanostructures enjoy the unique advantage of utilizing DNA as a structural scaffold for the precise generation of predicted nanostructures, while accurately labeling proteins to perform various functions 87. This synergy results in the development of novel hybrid nanomaterials with functionalities that cannot be achieved by individual biomolecules 87.

One noteworthy instance is the self-assembly of hybrid nanospheres made of DNA and streptavidin, which are highly versatile and can easily load chemotherapeutic drugs and functionalize with targeting molecules. In a one-pot self-assembly system, the nano-spheres go through three reaction phases 88. Doxorubicin (Dox), a popular cytotoxic chemotherapy drug, may be loaded into these nanospheres with ease 88. The loaded nanospheres, also called Dox-nanospheres, show traits of continuous drug release 88. Because of their exact modularity for *in vivo* imaging and cancer targeting, their biocompatibility, and their easy one-pot synthesis, self-assembled DNA-streptavidin hybrid nanospheres offer tremendous potential as a cancer-targeted nanoplatform 89.

#### Peptide based hydrogels

Peptide hydrogels have emerged as frontrunners in the realm of medical applications, owing to their remarkable structural and functional attributes. Numerous self-assembling peptides have been developed, holding promise as carriers for delivering anticancer drugs 90. These self-assembled peptides form hydrogels with nanotube-like structures. Extensive examinations have confirmed their mechanical robustness, stability, biocompatibility, and precise microscale dimensions. Additionally, these nanotubes exhibit thermal and chemical properties well-suited for their intended purpose 90.

An important milestone was reached by Mao et al., who pioneered a drug delivery system centered on a self-assembled peptide hydrogel. Their research successfully integrated two chemotherapeutic drugs, resulting in a notable enhancement of drug safety 91. This innovative device achieved controlled drug release through ester bond hydrolysis, showcasing its potential for precise and targeted anticancer drug delivery. Small peptide hydrogels are regarded as more advantageous for drug delivery due to their cost-effectiveness and customizable properties 92.

#### Collagen and gelatin

Collagen nanoparticles (collagen-NPs) are gaining recognition as promising biopolymeric nanoparticles due to their biodegradability and biocompatibility, characterized by low immunogenicity and non-toxicity. In a study, researchers isolated eight dis-tinct actinomycete strains from soil samples in Egypt, five of which demonstrated the ability to synthesize collagen-NPs 93. Among these, one strain, identified as Streptomyces *xinghaiensis* NEAA-1, exhibited the greatest biosynthetic potential, displaying anti-hemolytic, antioxidant, and cytotoxic properties for HCT116 cell lines. *In vivo* experiments indicated that collagen-NPs could suppress the growth of Ehrlich ascites carcinoma in mice, and when combined with doxorubicin (Dox), they achieved substantial tumor suppression 93.

Moving to another aspect, gelatin (degrade form of collagen) nanostructures loaded with paclitaxel offer controlled drug solubility both *in vitro* and *in vivo* settings, especially in aqueous environments, with a sustained release pattern 94. When administered intravesically, these paclitaxel-loaded nanostructures efficiently target bladder tumors while minimizing systemic absorption 94. Furthermore, these nanostructures maintain a consistent release of paclitaxel, addressing concerns related to drug dilution. This sustained release has the potential to reduce treatment frequency due to the prolonged maintenance of therapeutic drug levels 94.

#### Bacteria

Acoustic protein nanostructures, or gas vesicles (GVs), are aerated protein shells found in aquatic bacteria 95. These nanoscale structures enable strong ultrasound contrast, deep tissue penetration, and multimodal imaging. Genetically encoded GVs offer high-resolution, background-free imaging and can be customized for targeted applications 95. A group of researchers modified E. coli to gas vesicles (GVs) that target the tumor's hypoxic environment, enhancing ultrasound imaging and enabling focused ultra-sound ablation. Nanoparticles with IR780, perfluorohexane, and AQ4N exploited the hypoxic environment to boost therapeutic effects 96-98. GVs-*E. coli* accumulates in tumor regions, improving treatment precision while reducing side effects. The approach was validated through fluorescence, photoacoustic, and ultrasound imaging, demonstrating its diagnostic and therapeutic potential 99. Similarly, gas vesicles and functionalized GVs produced by cyanobacteria are used in sonodynamic therapy for cancer, enhancing ROS production and tumor growth inhibition 100. GVs' hollow structure improves ultra-sound contrast, functionalizes with dyes, and assists in imaging and therapy. *In vitro* and *in vivo* studies show that GVs effectively induce apoptosis and inhibit tumor growth while maintaining biocompatibility 100.

#### Soy protein

A study on soy protein isolate (SPI)-based nanoparticles used a pH-driven method to enhance curcumin's stability and bioavailability 101. SPI-Cur nanoparticles exhibited strong anticancer activity against HepG2 cells by triggering ROS-induced, mitochondria-mediated caspase apoptosis 101. biocompatibility 100.

#### Other protein nanostructures

Researchers explored stability, circulation, and tissue distribution of chemically self-assembled nanorings (CSAN) made from dihydrofolate reductase fusion proteins for potential *in vivo* applications 102. *In vivo* microPET/CT imaging revealed significant tumor accumulation and high-contrast imaging, demonstrating CSANs' potential for drug delivery and imaging 102. Protein based nanostructures enhance immune responses by targeting antigen-presenting cells and delivering therapeutic agents 57. Dual-targeting nanoparticles with monoclonal antibodies offer a stable, cost-effective alternative to bispecific antibodies, enabling more precise cancer treatment 103. Virus-like nanoparticles also show promise for antigen delivery in cancer immunotherapy 104. One study examined bacteriophage protein-based nanotubes as therapeutic nanocarriers 105. While colorectal cancer cells internalized them, primary macrophages cleared them, posing a challenge for therapy. Reduced macrophage clearance with age suggests potential for elderly cancer patients 105. Ferritin, a well-studied protein with an octahedral structure, can encapsulate drugs and target tumor cells via transferrin receptors 106.

### Limitations of protein nanostructures

However, using nanostructures in cancer therapy comes with challenges. Biological barriers can impede their efficient transport to target tissues, reducing delivery efficiency. Nanoformulations are also vulnerable to clearance by the reticuloendothelial system and often struggle with limited penetration into tumors compared to free drugs 107. Researchers have focused on developing functionalities to overcome these barriers and enhance tissue penetration 107. A multitude of factors may increase the risk of an unexpected loss of function or adverse effects, and the diverse range of nanomaterials may lead to intricate *in vivo* behaviours; therefore, careful consideration is necessary when developing multifunctional nanomaterials, considering the specific medical requirements as well as the unique properties of the materials 107. Another major limitation while using protein nanostructures is the difficulty adjusting its size and the high energies that it has due to its size 62. Drug delivery vehicles based on natural proteins have several benefits for treating cancer, but clinical translation is still a difficult task. Critical to therapeutic applications include concerns of large-scale synthesis, stability of formulations, *in vivo* distribution, metabolism, and excretion, as well as structural heterogeneity. To further understand nanostructure absorption and dispersion, sensitive *in vivo* detection methods are needed 61. In protein complexes, maintaining activity under mild conditions is crucial 108. Non-covalent or dynamic covalent strategies preserve structure and function 109,69 enabling reversible assembly to be like in nature 110. Chemical cross-linking, however, can limit protein dynamics due to stability constraints 111.

According to one study, (Wang et al., 2021) protein engineering suited to particular therapeutic applications and *de novo* design can overcome constraints associated with the functionalization of nanostructures based on the structures and properties of original proteins 61. Biotechnology enables tailored protein nanostructures, using genetic engineering and *de novo* design 112. Challenges remain in predictability, function integration, and avoiding aggregation 112.

## Green nanoparticles

**Figure 3:**
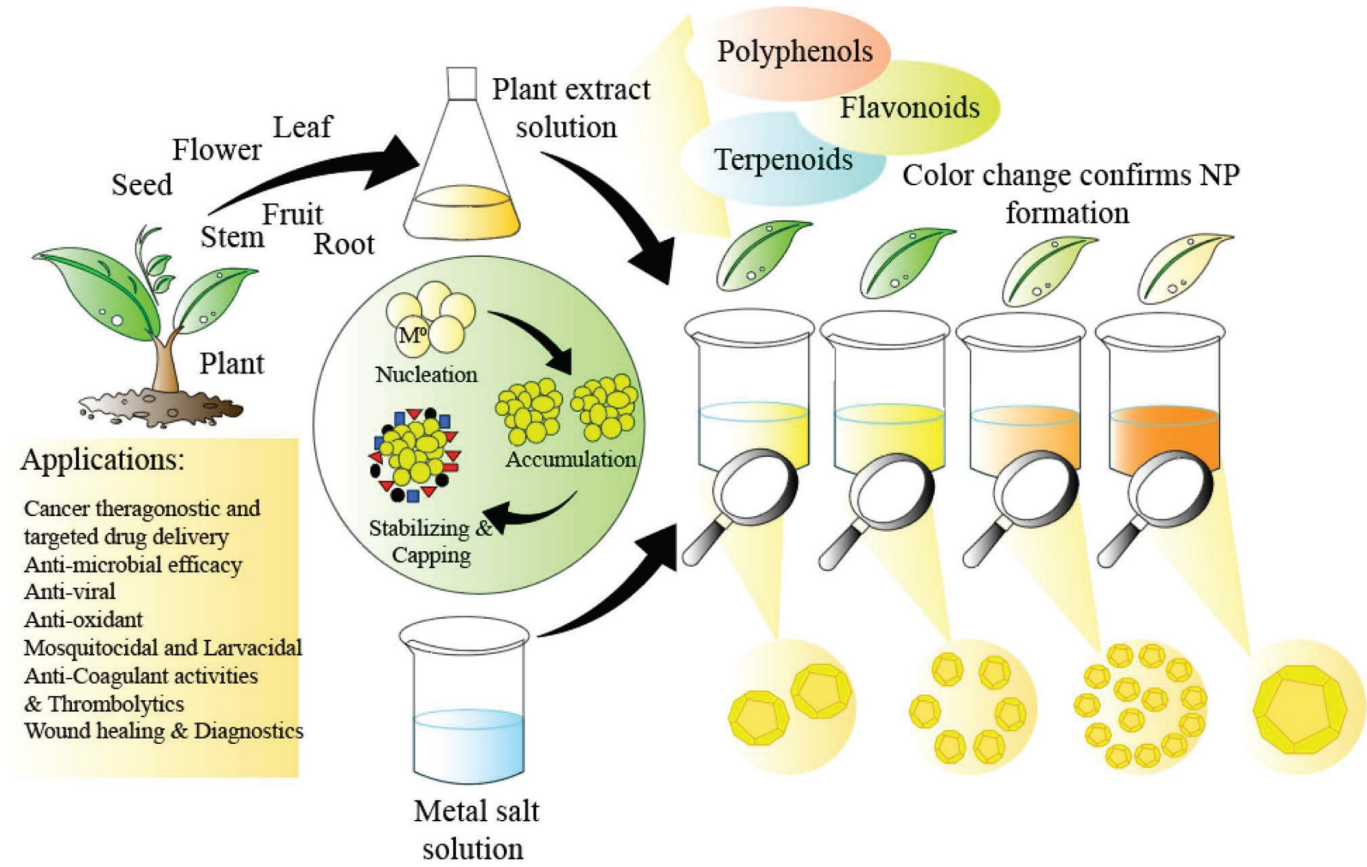
Thematic illustration of how green synthesized nanoparticles are formed 117.

For applications in biology and medicine where nanoparticle purity is paramount, the utilisation of natural resources to produce nanoparticles offers a sustainable, environmentally friendly, cost-effective, and chemical contaminant-free option 113. It is easy to mass-produce common and useful nanomaterials 113. There is no need for harmful chemicals in biological procedures. Plant extracts have less harmful byproducts that are easier to get rid of 113. Moreover, the green synthesis aims for better yields compared to the traditional chemical methods 114. The yields are mostly metal nanoparticles, and they are preferred for their unique optical, electronic, and catalytic properties 115. Green synthesis comes under the bottom-to-top approach synthesis process that uses chemical or biological methods, as compared to the top-to-bottom approach that uses physical methods 114. Nanoparticles can be fabricated biologically using plant extracts, which act as natural reducing agents 116. The process involves mixing dried, crushed plants with a solvent to obtain the extract, which is then combined with a metal ion solution 114. This green synthesis method eliminates the need for additional reducing agents as the plant extract facilitates the reduction of metal ions to nanoparticles 116,117 **(Figure 3*)***.

Favourable high yield requires the optimal temperature and pH, as temperature controls nucleation while pH controls the growth kinetics and stability 118,114. Nucleation is the first stage of nanoparticle formation, which determines the size and morphology of the product 114. Other conditions that also determine high yield are concentration of the reaction, reaction time and the plant extract components 114.

The advantages of using plant extracts are high as they contain natural polyphenols 119, carbohydrates, amino acids and proteins 120, which help in making stable nanoparticles with faster synthesis rate than nanoparticles not using plant extracts 121. Moreover, plant extracts serve as both reducing and stabilizing and capping agents, reducing aqueous solutions of metal salts into nanoparticles while preventing their aggregation over time 122,123. Green synthesized nanoparticles, such as those derived from curcumin, are also known to show dual properties of anti-cancer and anti-bacterial activity, in conjuction with silica-coated Fe3O4 magnetic nanoparticles 124.

### Green nanoparticles in cancer therapeutics

**Figure 4:**
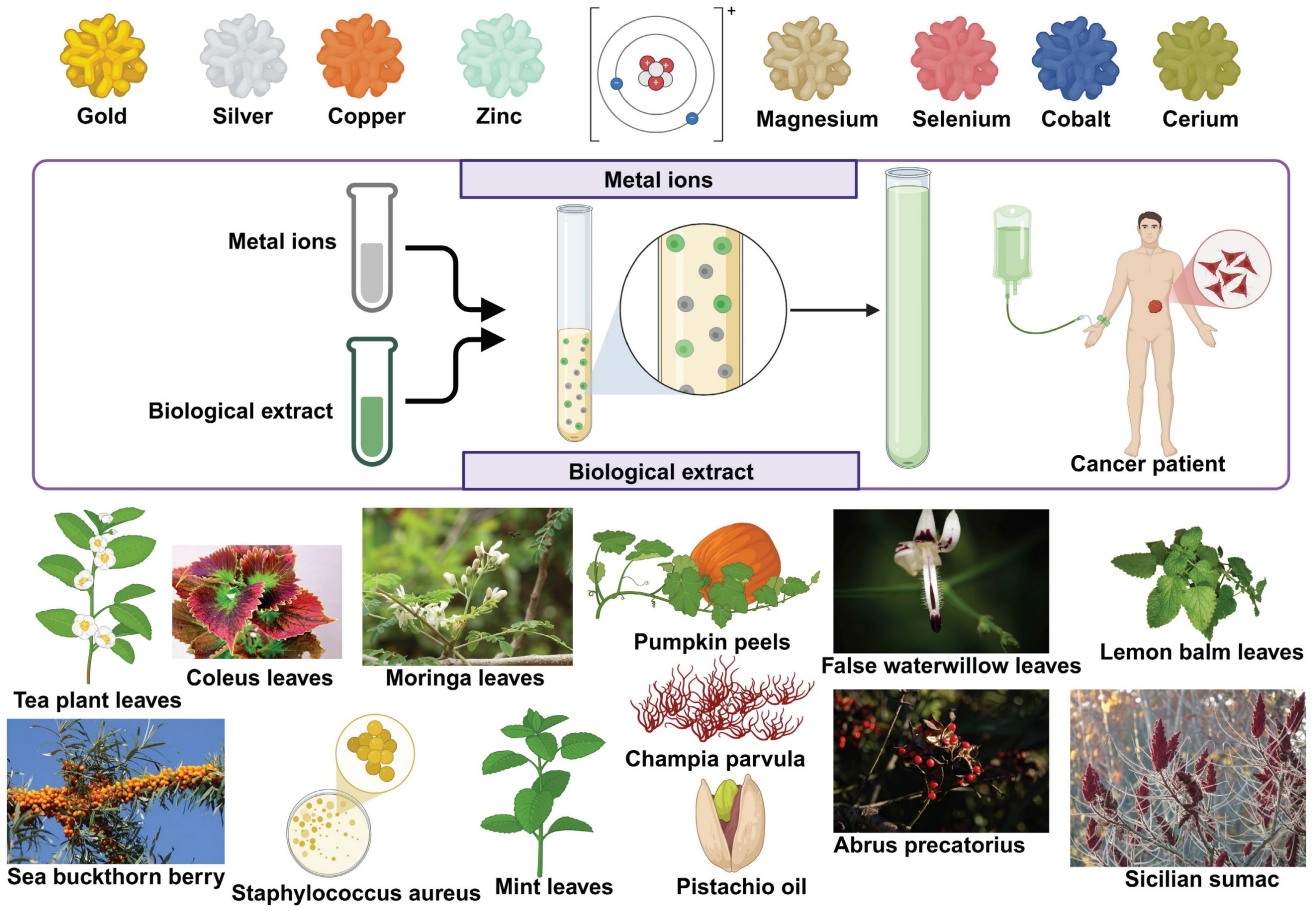
Different types of green synthesized nanoparticles, with their biological extract origin and metal ions showed.

There is a growing trend of green synthesis that focuses on creating nanoparticles with more stability 69. Comparing Nanoparticles made using green route synthesis to those made using physico-chemical techniques reveals that the former are more stable and effective 113. In the spirit of going green, be it with energy, or even with nanotechnology, researchers have found newer methods to design sustainable nanoparticles from renewable sources. There are different categories of nanoparticles that are green in synthesis, with metals from gold, silver, copper and so on ***(*Figure 4*)***.

#### Gold nanoparticles

Gold nanoparticles (AuNPs) have garnered increasing interest for their potential in bio-medical applications and the focus on green synthesis methods has intensified due to their associated biocompatibility and scalability. Previous studies have proved the anti-microbial activity of gold nanoparticles to increase with higher volume 125. A study by Ashikbayeva, et.al, describes the synthesis of AuNPs derived from green tea leaves and their subsequent application in the detection of the CD44 cancer biomarker via a biosensor constructed with ball resonator optical fibers 126. Characterized by its rapid and label-free operation, the biosensor can be fabricated in just 20 seconds and features a compact de-sign that holds promise for *in vivo* applications 126. In a study where gold nanoparticles (AuNPs) derived from the leaf extract of *Coleus scutellarioides* (L.) Benth was considered for breast cancer; the nanoparticles demonstrate effective free radical scavenging capabilities, particularly at higher concentrations compared to the plant extract alone 127. Furthermore, the cytotoxic effects of the AuNPs were evaluated against the MDA-MB-231 breast cancer cell line, revealing a dose-dependent reduction in cell viability for the cancer cells, while showing no significant cytotoxicity towards normal cells 127. Morphological changes in cancer cells, such as shrinkage and detachment, were observed after treatment, suggesting selective toxicity 127. In a study where gold nanoparticles (AuNPs) were synthesized using methanol extracts from Moringa oleifera seeds, there was an emphasis on an eco-friendly one-pot process 128. The antioxidant activity of the AuNPs was assessed using the DPPH radical stabilization method, and the nanoparticles exhibited a dose-dependent effect on A549 lung cancer cell proliferation showing anticancer property 128.

#### Silver nanoparticles

One such innovation is the green-synthesized silver nanoparticles (AgNPs) from *Cucurbita* spp. fruit peels 129, providing an environmentally friendly and cost-effective method. An experimentally synthesized Ag-NPs demonstrated a spherical morphology and stability, confirming their biocompatibility to be used as radiosensitizer for triple negative breast cancer 129. These nanoparticles led to increased expression of apoptotic-related genes in MDA-MB-231 cells and activated the apoptosis pathway and induced endoplasmic reticulum stress, which contributed to enhanced radiosensitization 129. Another study focused on the anticancer effects of silver nanoparticles synthesized from *Andrographis macrobotrys*, specifically targeting A549 lung cancer cells 130. The results demonstrated a pronounced dose-dependent increase in cytotoxic effects, with morphological changes induced in the treated cancer cells, revealing signs of cell shrinkage, membrane blebbing, and apoptotic surface formation 130. Another study evaluated the anticancer effects of silver nanoparticles derived from lemon balm leaves, graphene, and silver-graphene nanocomposites on MCF-7 breast cancer cells using the MTT assay to measure cell survival 131. Silver nanoparticles demonstrated significant cytotoxicity, notably increasing cancer cell death correlating with elevated reactive oxygen species and malondialdehyde levels, alongside decreased glutathione 131. These mechanisms promoted apoptosis in breast cancer cells 131. In another landmark study, the antioxidant potential of AgNPs synthesized from *Moringa peregrina* leaf extract, showed strong cytotoxicity activity against MCF-7 breast cancer cells 132. The AgNPs induced cell death primarily through apoptosis rather than necrosis, influenced by oxidative stress from reactive oxygen species and modulation of key signaling pathways like the p53 gene 132. Additionally, AgNPs was found to impede tumor migration and angiogenesis, potentially reducing the risk of metastasis in cancer treatments 132. A group of researchers synthesized silver nanoparticles from red seaweed *Champia parvula* extract, leveraging its antioxidant-rich phytochemicals like phenols, flavonoids, and tannins 133. These *Champia parvula*-mediated AgNPs (Cp-AgNPs) showed stability and antimicrobial activity against *Streptococcus mutans*, *Staphylococcus aureus*, and *Candida albicans*, and anticancer effects, especially against lung cancer cells indicating potential as multifunctional cargo 133.

#### Copper nanoparticles

Synthesis of copper oxide nanoparticles for cancer therapy is also an underexplored area, and recently, significant advancements have been made in utilizing copper oxide nanoparticles (CuONPs) for gene delivery applications 134. Looking at a study that confirmed the stability of conjugated copper oxide nanoparticles (CuONPs) with folate, there was significant cytotoxicity observed against MDA-MB-231 breast adenocarcinoma cells by induced apoptosis and reactive oxygen species. The nanoparticles were synthesized using Staphylococcus aureus extracts 135.

There are studies where copper nanoparticles were characterized for anti-microbial activity 136 and were tested against seven strains of microbes, with the right sized nanoparticles, and fast synthesis 137. In a recent study, CuONPs were biologically synthesized using leaf extract from Melia azedarach, followed by functionalization with chitosan and polyethylene glycol 134. The CuONPs were then conjugated with folate as a targeting ligand to enhance their specificity for cancer cells 134. *In vitro* studies assessing cytotoxicity showed that the CuONPs maintained cell viability greater than 70% across various cell lines, including human embryonic kidney (HEK293), breast adenocarcinoma (MCF-7), and cervical cancer (HeLa) cells 134.

Another study explored the phytochemical-assisted synthesis of copper nanoparticles (CuNPs) using the stem extract of Hippophae *rhamnoides*, a plant indigenous to the Himalayas and known for its rich phytochemical profile 138. Further investigation into the CuNPs' anticancer potential was conducted on HeLa cell lines 138. Results from the MTT assay indicated a dose-dependent cytotoxic effect with an IC50 value of 48 µg/mL, underscoring a strong inhibitory impact on cancer cells 138.

#### Zinc nanoparticles

Cytotoxicity assays conducted on HT-29 colon cancer cells revealed that ZnO(Zinc Oxide) nanoparticles synthesized with Artocarpus hirsutus seed extract exhibited potent dose-dependent cytotoxic effects 139. The nanoparticles triggered reactive oxygen species generation, leading to oxidative stress that damaged cellular structures and pathways critical for cell survival 139. This oxidative environment contributed to the downregulation of the anti-apoptotic Bcl-2 gene, promoting apoptosis in cancer cells 139. Additionally, the study highlighted morphological changes associated with apoptotic cell death, supporting the mechanism of action 139. The study by Mongy et. al investigated the biogenic synthesis of zinc oxide nanoparticles (ZnO NPs) using Rhus *coriaria* fruit extracts, demonstrating their eco-friendly production and potent anti-cancer effects on breast cancer cells, MCF-7 and MDA-MB-231 140. Mechanistic studies indicated that ZnO NPs induce apoptosis in MDA-MB-231 cells, as evidenced by significant nuclear fragmentation, increased apoptotic populations, and S-phase arrest 140. Additionally, the ZnO NPs significantly hindered the colony-forming ability of MDA-MB-231 cells and their wound healing capabilities, indicating promising anti-migratory properties 140. Zinc nanoparticles also exhibit anti-microbial activity, causing bacterial cell death as soon as they interact, and the process has been experimentally known to be the simplest, in comparision with other nanoparticles 141.

#### Magnesium nanoparticles

Magnesium oxide nanoparticles (MgO NPs) are garnering increasing attention compared to other metal oxide nanoparticles due to their unique properties and diverse applications 142. Their enhanced stability-to-weight ratio, lightweight nature, and recyclability make them particularly appealing for various fields, especially in biological applications 142. MgO NPs are nontoxic and hygroscopic, which further contributes to their utility in bio-medical contexts 142. In a study, the synthesis of magnesium oxide nanoparticles utilizing the bark extract of Abrus *precatorius* demonstrated high efficacy of treatment with MgO NPs inducing both apoptosis and necrosis in a concentration-dependent manner 143. Cytotoxicity investigations revealed the cytotoxic effects of MgO NPs were time- and dose-dependent 143. Notably, the MgO NPs induced reactive oxygen species formation, leading to DNA damage and subsequent apoptosis in the A375 cell line 143. Magnesium nanoparticles have additional anti-microbial activitiy against food-borne pathogens 144.

#### Other metallic nanoparticles

In recent years, spinel ferrite nanoparticles have garnered significant attention for their promising applications in biomedicine, particularly in cancer treatment through plasmonic photothermal therapy 145. This innovative approach utilizes nanoparticles that exhibit strong absorption in the infrared region, enabling localized heating upon laser irradiation 145. Among various spinel ferrite nanoparticles, cobalt ferrite (CoFe₂O₄) and zinc ferrite (ZnFe₂O₄) have emerged as notable candidates due to their unique magnetic and thermal properties 145. The efficacy of these biosynthesized nanoparticles was evaluated against MCF-7 breast cancer cells in conjunction with laser radiation; revealing a significant reduction in cancer cell viability, with CoFe₂O₄ nanoparticles exhibiting greater photothermal efficacy compared to ZnFe₂O₄ 145. Both nanoparticles showed acceptable biocompatibility with normal cells, emphasizing their potential for safe biological applications 145. The cytotoxicity mechanism is primarily attributed to the generation of reactive oxygen species, inducing oxidative stress and disrupting cellular functions, while ZnFe₂O₄ nanoparticles demonstrated enhanced efficacy in inducing cell death, likely due to zinc's role in cellular metabolism and tumor suppression pathways 145. Another promising anti-cancer study investigated the synthesis of cerium oxide nanoparticles (CeO2 NPs) utilizing pistachio Vera Pericarp essential oil as a coating, focusing on human prostate (LNCap) and breast cancer (MCF7) cell lines 146. The biological assays demonstrated that CeO2 NPs exhibited significant cytotoxic effects on LNCap and MCF7 cells, with a marked decrease in cell viability observed when used in conjunction with zoledronic acid 146. These nanoparticles affected cell proliferation, apoptosis, and migration by regulating the expression of apoptosis-related genes BCL-2 and BAX, as evidenced by real-time PCR analysis 146. Specifically, treatment with CeO2 NPs resulted in a reduction of BCL-2 expression and an increase in BAX expression, thereby promoting apoptosis 146. The results indicate that CeO2 NPs, especially when combined with ZA, could be effective therapeutic agents for treating prostate and breast cancers 146. The anticancer properties of selenium nanoparticles were evaluated using human breast cancer MCF-7 cell lines 147. At a concentration of 500 mg/mL, SeNPs significantly reduced cell viability, lowering it to 61.2 ± 2.2% after 24 hours of exposure 147. This indicated a substantial cytotoxic effect on cancer cells, suggesting that SeNPs could potentially inhibit cancer cell proliferation 147. The results highlight the potential of SeNPs as a promising agent for cancer treatment, especially for breast cancer, where targeted nanoparticle therapies are of growing interest 147. Researchers synthesized pure and Cobalt-doped Nickel Oxide nanoparticles (1%,3%, and 5% Co-NiO-NP) using Salvadora persica plant extract 148. Physicochemical analyses confirmed uniformly spherical nanoparticles at nanoscale. Cytotoxicity tests on MCF-7(breast cancer) and HUVEC (human endothelial) cell lines showed that Co-doped NiO-NP had greater inhibitory effects than pure NiO-NP, with cytotoxicity increasing with cobalt content 148. These findings support the potential of Co-doped NiO nanoparticles for biological applications, particularly in cancer treatment 148. A study that examined the effects of Mentha spicata-loaded Fe nanoparticles on LS174t colon cancer cells revealed changes in the expression of pro-apoptotic BAX and anti-apoptotic Bcl2, suggesting a pro-apoptotic impact from the combination of Mentha spicata and Fe nanoparticles 149. The synthesized nanoparticles demonstrated note-worthy interactions with LS174t cells, showing not only significant cytotoxicity but also alterations in the apoptotic pathway, as indicated by the modulation of BAX and Bcl2 expression 149. This pro-apoptotic activity, particularly pronounced in the Mentha spicata-loaded Fe nanoparticles, suggests their potential role in enhancing the effectiveness of existing cancer therapies, especially when combined with radiotherapy 149.

### Limitations of Green nanoparticles

There is no doubt in establishing the fact that green nanoparticles are sustainable, efficient and have additional properties ***(*Table 1*)***, however, there are certain limitations 150. There are risks of off-target toxicity as nanoparticles tend to translocate across different barriers with ease 150. The extent of biochemical reactions caused by the nanoparticles within the human body still needs complete comprehension 150. Unregulated translocations can result in harmful effects such as oxidative stress, cytotoxicity and genotoxicity 150. Moreover, sourcing of raw material is sometimes challenging in green synthesis as some natural sources tend to be endemic to particular regions 116. Long reaction time and consequential energy consumption is also a matter of concern when it comes to the synthesis process 116. Variable outcomes in terms of particle size, quality and effectiveness are potential issues that can happen during the synthesis process 116. Since green synthesis can depend on a lot of factors such as pH, temperature and bio-chemical conditions, aggregation is a possibility that can hinder drug efficiency 151. Proper stabilization and guidance system for green nanoparticles within the body can make a lot of difference in cancer theranostics.

## Recent research heralding protein-green nanoparticle nanosystem specific for cancer theranostics

**Figure 5:**
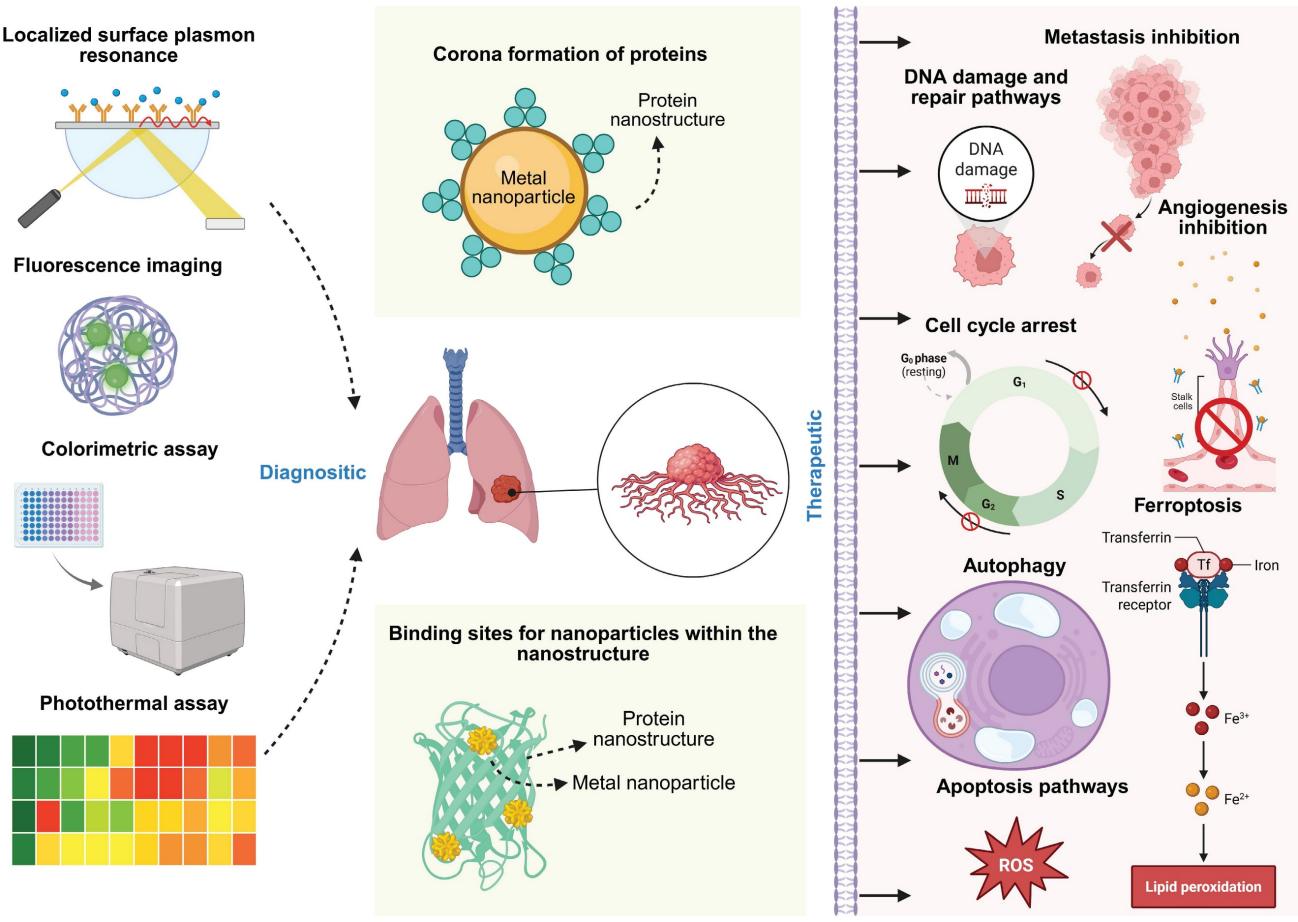
The overall mechanism of diagnostic and therapeutic nano systems made of protein and metal nanoparticle.

**Figure 6:**
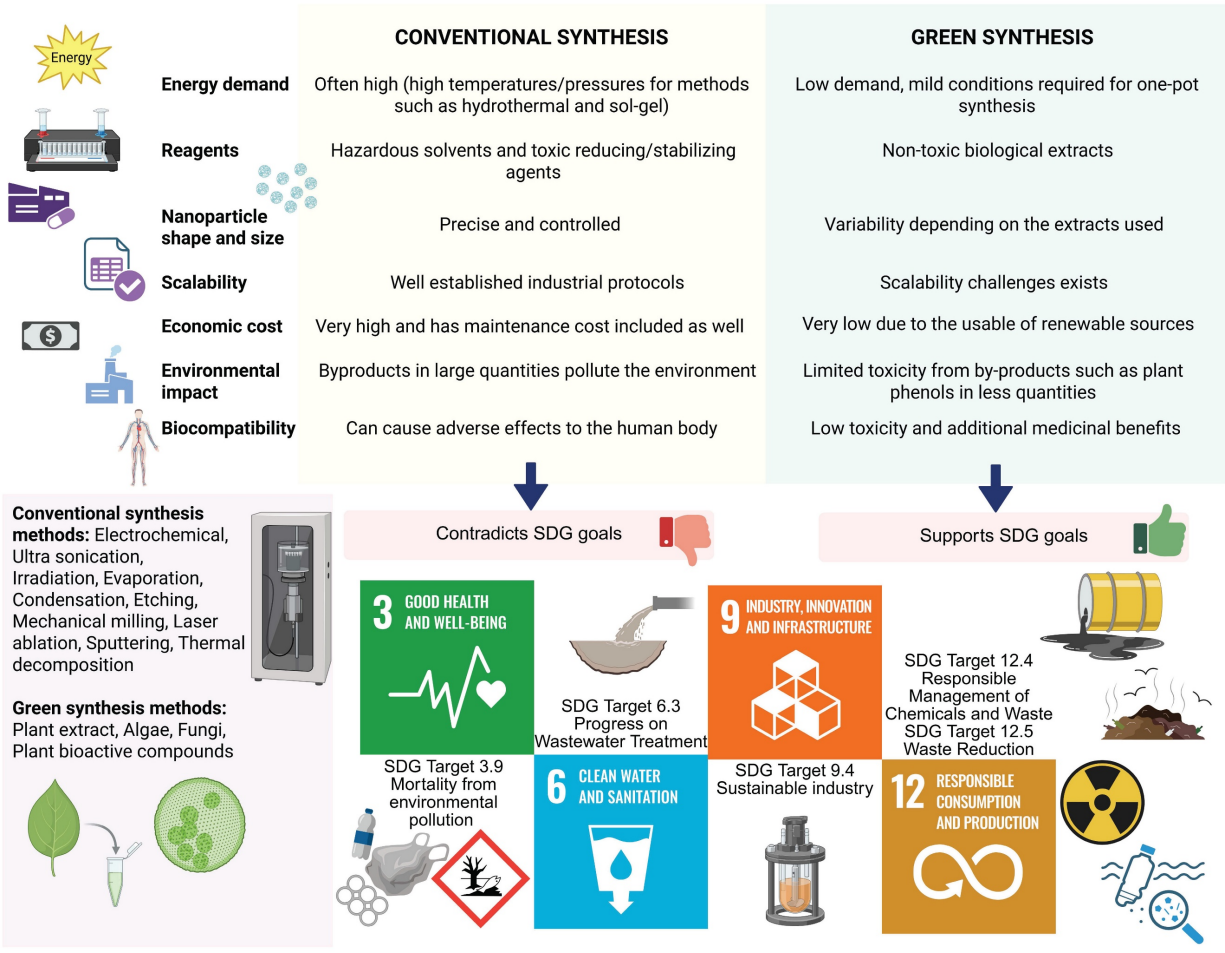
Advantages and disadvantages of green synthesis methods over conventional methods, in line with UN's sustainability goals for 2030. The sustainable development goals that are mentioned in this context include SDG 3: Good health and well-being, SDG 6: Clean water and sanitation, SDG 9: Industry, Innovation and Infrastructure and SDG 12: Responsible consumption and production, all of which support the need of designing biosynthesized nano systems from protein and green synthesis origins 167.

Latest approach involves the use of nanogels made of β-Lactoglobulin to be used as cargo carriers for metal nanoparticles 152,153. Compared to the pristine nanoparticles, the absorption rate is higher when the metal nanoparticles are conjugated with the nano-gel, expanding the utility of the nanoparticles. Moreover, protein based nanogels can help with easier cell penetration and can be eviscerated by phagocytosis 152,154. Silver nanoparticles synthesized from *clitoria ternatea* plant extract combined with sodium alginate and gelatin polymer blends has shown apoptotic activity in lung cancer cell line 155. Gold nanoparticles derived from *Cassia fistula* had antimicrobial and anticancer activity against E. coli DH5-Alpha and skin melanoma cell line, while maintaining good stability conjugated with Human serum albumin 156. *Tricholoma crassum* derived gold nanoparticles with natural protein coating that show anti-microbial activity against multiple microbes, while selectively binding to sarcoma cells to induce apoptotic activity 157. The area of protein-green nanoparticle nanosystems is still an area that needs more exploration, particularly conducting *in vivo* studies and further clinical studies. This biological based method is generally simple and reduces the amount of chemicals used for synthesis. The protein component acts as a stabilizing and reducing agent for the metal nanoparticles, with a focus on targeted drug delivery 158. Additionally, there is a positive effect of proteins on the synergistic nanoparticle in maintaining the colloidal stability of the nanoparticle 159. The protein's corona often has additive properties such as accumulation, degradation, inflammation, cellular uptake and clearance, which makes the nano system compatible with complex organism systems like that of human 159. Nanoparticle specific processes such as glass transition, crystallization, gelation and flocculation which can be influenced by the protein involved 159. This overall can control the biological reactivity of the nano system, making it safer and sustainable 159. Design principles state that the nano systems can be used in diagnostics through fluorescence imaging, colorimetric assay, photothermal and localized surface plasmon resonance 160. They could also cause cell cycle arrest, apoptosis, and cause the tumour to be treated 161** (Figure 5)*.*** One of the recent papers talk about green synthesized zinc nanoflowers from *Heliotropium indicum* extract, coated with albumin being used to induce oxidative stress in melanoma cells 162. Multifunctionality is possible due to material synergy, the principle behind effective stimuli response for the hybrid nanosystems 163. *In vivo* research faces limitations such as large-scale manufacturing, and *in vitro* studies help in detailed testing in controlled settings 164. Reproducibility is another challenge due to the involvement of organic compounds 164. As most of them are usually one pot approaches, they do not create toxic byproducts 165. However, there are chances of minimal toxicity due to incorrect concentrations, excess metal ions, excess phenols or salts 165. Nevertheless, the green synthesis methods have an environmental advantage over the conventionally synthesis methods, which is in line with the United Nations's SDGs (Sustainable Development goals) for 2030 166,167,168** (Figure 6)**. The United Nations had envisioned 17 goals and 169 targets as part of a blueprint to develop sustainable practices for living 167. Biosynthesized nano systems are the need of the hour as they sustain major SDG targets such as Good health and well-being (SDG 3), Clean water and sanitation (SDG 6), Industry, Innovation and Infrastructure (SDG 9) and Responsible consumption and production (SDG 12) 167.

## Future prospects of cancer theranostics: safe and efficient protein-green nanoparticle nanosystems

**Figure 7:**
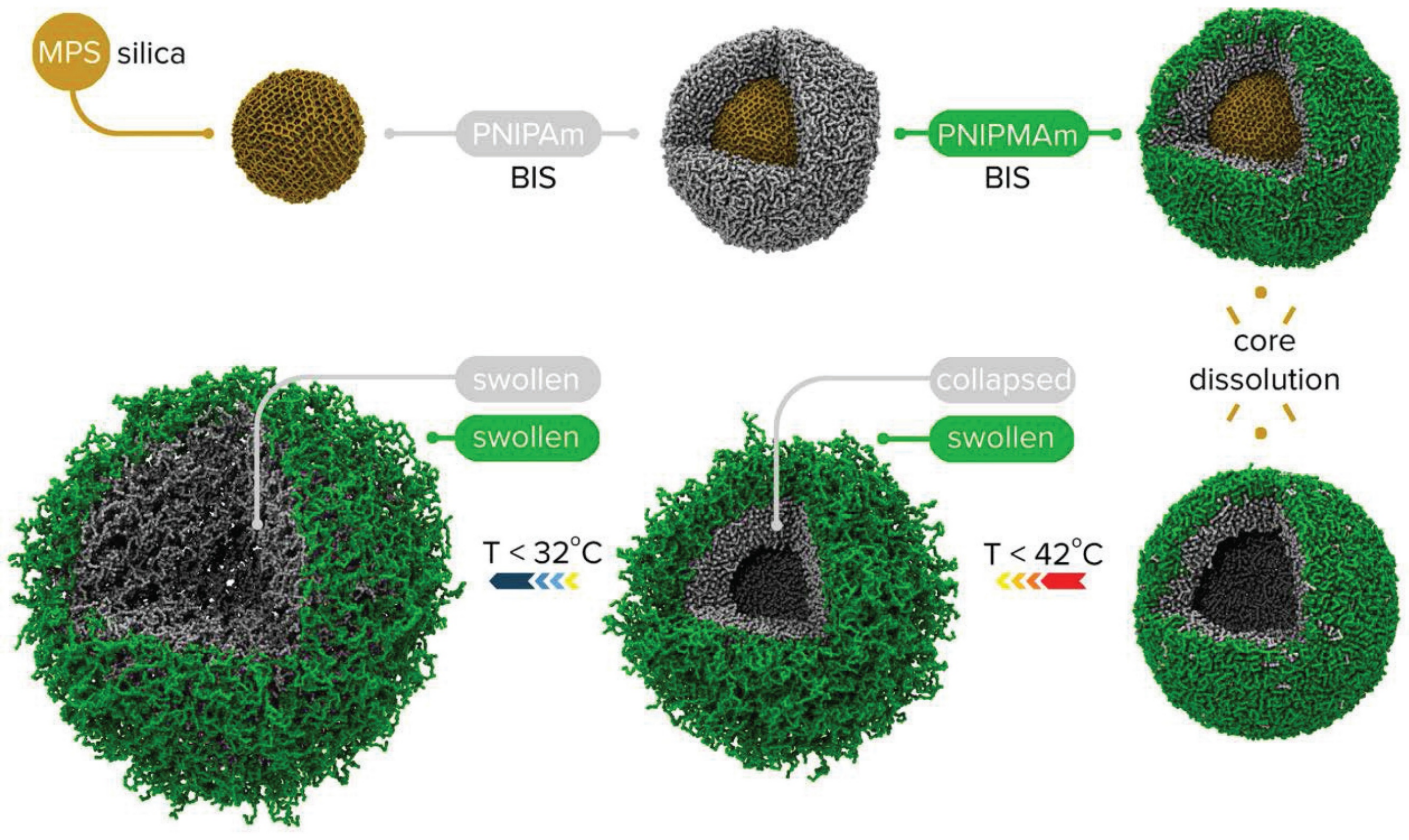
Example of core shell and hollow shell nanogels being synthesized 179.

Nanoparticles derived from green synthesis is an eco-friendly, cost-effective method and utilizes microbes and plants, leveraging their ability to absorb and transform inorganic metal ions 169. Compared to traditional synthesis methods, green synthesis is more sustainable and biologically safe 169. A lot of invitro studies are present that strongly establish the anti-microbial and anti-cancer activity of the green synthesized nanoparticles. However, their full potential could be realized through more *in vivo* studies, especially with their conjugation with protein nanostructures. There are many studies that have proved the anti-cancer and anti-bacterial activities of protein-green nanoparticle nanosystems, where the nanoparticles probably were not green synthesized. These nanosystems come under the class of hybrid nanostructures, which combines different nano materials, enhancing their functionality through unique synergistic properties 170. They could be core-brush nanoparticles, hybrid nanogels or core-shell nanoparticles, based on the configuration of the nanomaterial produced ***(*Figure 7*)*** 171. The future research can focus on fine tuning the properties of the nanosystems 172, improve multi-functionality 22, and enhance their native properties 22. The nanosystems can be designed through sol-gel, solution-phase or through ligand exchange 170. To make the process easier, artificial intelligence can be used during the research and development process for prediction, modelling, material discovery and design 173. Using Nano-Quantitative structure-activity relationship (NQSAR) principles, Artificial intelligence and nanotechnology can be brought together for structural characterization as well as toxicity prediction 174. Artificial neural networks can be used for QSAR, while CORAL can be used for cell viability tests and toxicity prediction 174-177. Support vector machines can be used for target specification while Logistic linear regression can be used for Adverse outcome pathways 174,177,178. Nanoinformatics could be leveraged to reduce the time spent in research and development, generating strong candidates for *in vivo* and pre-clinical studies. The future should focus on establishing a comprehensive pipeline for entirely naturally synthesized nanosystems, minimizing reliance on artificial nanomaterials. This approach would help protect the environment while offering a safer, side-effect-free strategy to alleviate the burden of cancer on humans.

## Conclusion

In this review, different types of protein-based nanomaterials and green synthesized nanoparticles are discussed along with their potential in cancer theranostics. The review highlights the promising applications of protein-based nanostructures and green nano-particles in cancer therapy, focusing on their multi-capabilities against cancer and bacterial infections while emphasizing the need for further research to optimize their therapeutic effectiveness. More *in vivo* studies need to be done as part of future research to create a class of hybrid and efficient nanosystem that is sustainable and safe for humans.

## Figures and Tables

**Table 1:** Multifunctionality of green nanoparticles derived using green synthesis

Element	Bioextract origin	Anti-microbial	Anti-oxidant	Anti-proliferative	Anti-inflammatory	Source
Gold	Coleus scutellarioides	-	+	-	-	Al-Mafarjy et al., 2024
Gold	Moringa oleifera	+	+	+	-	Bouttier-Figueroa et al., 2024
Silver	Andrographis macrobotrys Nees	+	+	-	+	Sivakumar et al., 2023
Silver	Melissa officinalis	-	+	+	-	Motafeghi et al., 2023
Silver	Moringa peregrina	-	+	-	-	Al Baloushi et al., 2024
Silver	Champia parvula	+	+	-	-	Viswanathan et al., 2024
Zinc	Artocarpus hirsutus	+	-	-	-	Sampath et al., 2023
Magnesium	Abrus precatorius	+	+	-	-	Ali et al., 2023

## Abbreviations

DNA: Deoxyribonucleic acid
FDA: Food and Drug Administration
GP60: Glyco Protein 60
HAS: Human serum albumin
HER2: Human Epidermal Growth Factor receptor 2
Zn-CSNPs: Zinc-Chitosan nanoparticles
HEK 293T: Human Embryonic Kidney 293 T antigen
MUC1: Mucin 1
HCT116: Human Colorectal carcinoma cell line
microPET/CT imaging: Micro Positron Emission Tomography/Computed Tomography imaging
QSAR: Quantitative structure-activity relationship
